# The Effect of an External Magnetic Field on the Electrochemical Capacitance of Nanoporous Nickel for Energy Storage

**DOI:** 10.3390/nano9050694

**Published:** 2019-05-04

**Authors:** Haixia Zhang, Zhifei Han, Qibo Deng

**Affiliations:** Tianjin Key Laboratory of Advanced Functional Porous Materials, Institute for New Energy Materials and Low-Carbon Technologies, School of Materials Science and Engineering, Tianjin University of Technology, Tianjin 300384, China; haixia0202@126.com (H.Z.); 183124331@stud.tjut.edu.cn (Z.H.)

**Keywords:** magnetic field, nanoporous nickel, specific capacitance, electrochemical energy storage

## Abstract

This work investigates the effect of a magnetic field on the electrochemical performance of nanoporous nickel (np-Ni). We first compare the electrochemical capacitance of np-Ni electrodes, which were prepared using the chemical dealloying strategy under different magnetic flux densities (B = 0, 500 mT). Our experimental data show that np-Ni_500_ prepared under an external magnetic field of 500 mT exhibits a much better electrochemical performance, in comparison with that (np-Ni_0_) prepared without applying a magnetic field. Furthermore, the specific capacitance of the np-Ni_0_ electrode could be further enhanced when we increase the magnetic flux densities from 0 T to 500 mT, whereas the np-Ni_500_ electrode exhibits a stable electrochemical performance under different magnetic flux densities (B = 0 mT, 300 mT, 500 mT). This could be attributed to the change in the electrochemical impedance of the np-Ni_0_ electrode induced by an external magnetic field. Our work thus offers an alternative method to enhance the electrochemical energy storage of materials.

## 1. Introduction

One of the effective strategies for electrochemical energy storage, the supercapacitor, has been popularly studied due to its high power density and long lifecycle [1,2,3]. There are two different well-known mechanisms for the charge storage of electrochemical supercapacitors. The first one is the electrochemical double-layer behavior. The second one is the pseudo-capacitor, in which the electrochemical energy storage comes from fast and reversible electron-exchange reactions at or near the electrode surface [4,5]. The electrode materials of a supercapacitor mainly include carbon materials [6], conductive polymers [7], metal oxides, and their composites [8]. Nickel is one of the popular materials used as the supercapacitor electrode due to its high pseudo-capacitance performance [9,10,11]. Recently, researchers have employed materials with a nanoporous structure because of this structure’s high specific surface area and high porosity [12,13]. However, nickel metal, one of the ferromagnetism materials, is hard to form into a perfect nanoporous structure due to its strong agglomeration [14].

In the past, attention was focused on modulating the composition, structure, and morphology of active materials to improve the energy storage performance of an electrode. Recently, there has been increasing interest in the strong coupling between electrochemical performance and an external physical field, e.g., between mechanical methods [15,16] and a magnetic field [17,18]. The elastic strain on the electrode surface, as one of the simple mechanical methods, can be used to modulate the reactivity of electrodes. Such an effect of strain can be understood to induce change in the crystal lattice of the electrode material or to change the free energy in the metal–electrolyte system [16,19]. Additionally, an external magnetic field has been widely used during the preparation of materials for application in the fields of electrocatalysis [20] and energy storage [21,22,23]. For example, a magnetic field can control the growth of 2 × 2 tunnel MnO_2_ nanostructures for capacitance enhancement [23]. The external magnetic field can also directly improve the electrochemical performance of materials. Zeng et al. demonstrated that an external magnetic field can improve the specific capacitance of MnO_2_/electrospun carbon nanofibers (ECNFs) due to the magnetic-enhanced transportation of cations in the electrolyte [24]. In this aspect, the magnetic field influences the transport ability of the charge and modulates the charge density gradient to improve the electrochemical performance. This is well-known as the effect of the Lorentz force [24,25,26].

We confirmed that an external magnetic field strongly affects the morphology of nanoporous nickel (np-Ni) in our previous work [27]. The finer nanostructure could be perfectly prepared by adjusting the applied magnetic flux density. It is still not clear, however, whether the magnetic field can influence the electrochemical performance of np-Ni. Based on previous findings, this study includes two main parts: (i) an investigation of the electrochemical energy storage performance of np-Ni for different dealloyed samples which were prepared without a magnetic field or under the magnetic flux density of 500 mT; (ii) a further investigation of the electrochemical performance of different np-Ni samples under the effect of an external magnetic field with different magnetic flux densities (0, 300, and 500 mT). The results illustrate that the magnetic field can be used as one of the interesting and simplest strategies to enhance the electrochemical energy storage of electrode materials.

## 2. Materials and Methods

### 2.1. Materials

The experimental materials used in this work were KOH powder (Shanghai Titan Scientific Co., Ltd., Shanghai, China), NaOH powder (Shanghai Titan Scientific Co., Ltd.), H_2_SO_4_ electrolyte (Sinopharm Chemical Reagent Co., Ltd., Shanghai, China, 98%), acetone solution (Sinopharm Chemical Reagent Co., Ltd.), ethanol (Hengshan Chemical Technology Co. Ltd., Tianjin, China, 99.7%), polyvinylidene fluoride (PVDF, Tianjin Aiweixin Chemical Technology Co., Ltd., Tianjin, China), *N*-methyl-2-pyrrolidinone (NMP, Adamas Reagent Co., Ltd., Shanghai, China), acetylene black (Hefei Kejing Materials Technology Co., Ltd., Hefei, China), and nickel foam (Tianjin Metal Material Company, Tianjin, China).

### 2.2. Electrode Preparation

The experimental details on the preparation of the nanoporous Ni were described in our previous work [25]. We present a brief display for a self-contained description here: firstly, the Ni_20_Al_80_ ribbons were prepared by smelting and the melt-spinning method. Secondly, the Ni_20_Al_80_ ribbons were dealloyed in an aqueous solution of 2 M NaOH at room temperature to remove the Al element from the alloy bulk and then to form a nanoporous structure of np-Ni. The chemical dealloying process was performed under two different conditions: (i) without an external magnetic field (denoted as np-Ni_0_) and (ii) under a magnetic flux density of 500 mT (denoted as np-Ni_500_). 

The Ni foam was washed with H_2_SO_4_ (1 M), acetone, and pure water to remove impurities from the surface and it was then dried at 60 °C. The as-prepared np-Ni, acetylene black, and PVDF were mixed with the ratio 8:1:1. This mixture was dispersed in NMP solvent and was ground thoroughly to form a slurry using a mortar. The slurry was then spread onto Ni foam (1.0 cm × 1.0 cm), and dried under vacuum at 60 °C for 12 h.

### 2.3. Electrochemical Measurements and Materials Characterization

The electrochemical measurements were performed with a three-electrode system which used np-Ni samples as the working electrode, a carbon rod as the counter electrode, a commercial Ag/AgCl electrode as a reference electrode, and 1 M KOH as the electrolyte. The electrochemical measurements were carried out using the CHI 760E electrochemical workstation (Shanghai, China). Cyclic voltammetry (CV) was performed in the potential window between 0 and 0.55 V (vs. Ag/AgCl) at room temperature at different potential scan rates. Galvanostatic charge–discharge measurements were performed at different current densities in the potential window between 0 and 0.55 V at room temperature. Electrochemical impedance spectra (EIS) were obtained in a frequency range from 0.1 Hz to 100 kHz with an alternating current (AC) amplitude of 5 mV.

The composition of specimens was characterized by an X-ray diffractometer (XRD, Rigaku D/max-2500, Tokyo, Japan) with Cu Kα radiation. The morphology of np-Ni was characterized by a scanning electron microscope (SEM, Quanta FEG 250, Hillsboro, OR, USA). The specific surface area was examined by the Brunauer–Emmett–Teller method (BET, Quantchrome, Autosorb-iQ-3, Boynton Beach, FL, USA).

## 3. Results and Discussion

### 3.1. The Comparasion of Electrochemistry Performances of Different Nanoporous Ni Samples

**Figure 1:**
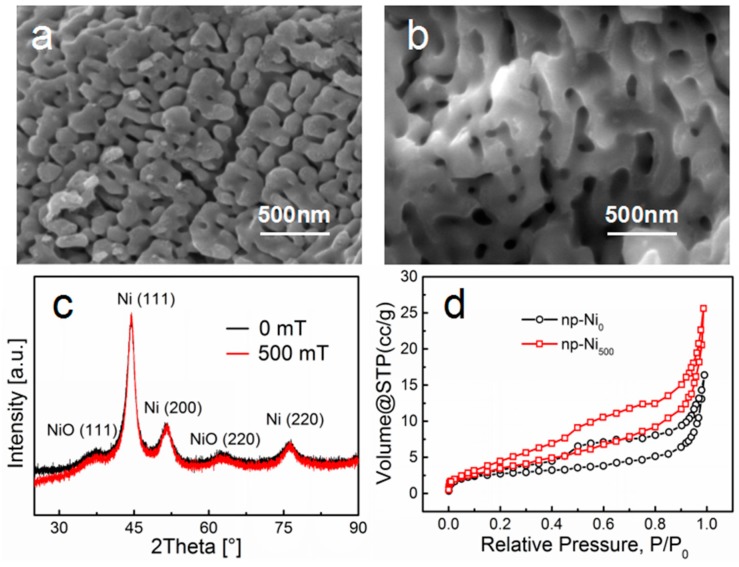
SEM images of (**a**) np-Ni_0_ and (**b**) np-Ni_500_. (**c**) XRD patterns of different np-Ni samples. (**d**) Nitrogen adsorption–desorption isotherms of np-Ni_0_ and np-Ni_500_.

We previously investigated the significant effect of an external magnetic field on the chemical dealloying process of Ni–Al alloy, giving rise to different morphologies. The morphologies of the np-Ni samples are also presented in this work by SEM, as shown in Figure 1a,b. The morphology of np-Ni_500_ obtained under the magnetic intensity of 500 mT exhibited a better 3D porous structure than that (np-Ni_0_) without applying an external magnetic field. In comparison with the morphology of np-Ni_0_, the external magnetic field of 500 mT made the ligament of np-Ni_500_ finer and more homogeneous. Figure 1c shows the XRD patterns of the np-Ni_0_ and np-Ni_500_ samples. The diffraction peaks correspond to the (111), (200), and (220) crystal planes of Ni metal. Since the dealloying process was performed in the electrolyte without Ar gas bubbling, the NiO phase can be detected on the XRD patterns. The diffraction peaks are attributed to the (111) and (220) crystal planes of NiO. From the SEM images and XRD results, the magnetic field affects the morphology of the nanoporous material during the dealloying process but has no effect on changing the phase composition. This agrees with our previous work [27]. Figure 1d shows the nitrogen adsorption–desorption isotherms for the samples. The isotherms show type IV hysteresis loops, which indicate the mesoporous nature of the samples. The BET surface area was 8.9 m^2^g^−1^ for np-Ni_0_ and 15.4 m^2^g^−1^ for np-Ni_500_. Thus, the np-Ni_500_ sample exhibited a larger surface area. We compared the pore size distributions of np-Ni_0_ and np-Ni_500_ samples by contrasting the SEM images (see Figure S1, Supplementary Materials). The average pore size was 70 ± 19 nm for np-Ni_0_ and 100 ± 28 nm for np-Ni_500_. The average pore diameter of np-Ni_500_ sample was larger than that of np-Ni_0_.

**Figure 2:**
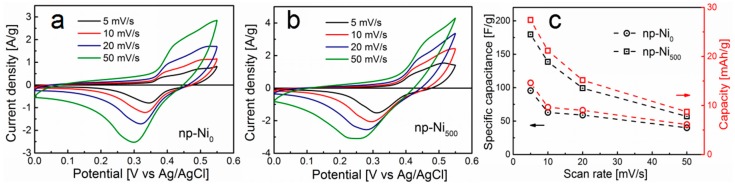
In a 1 M KOH aqueous electrolyte, cyclic voltammetry (CV) curves of (**a**) np-Ni_0_ and (**b**) np-Ni_500_. (**c**) The specific capacitance at corresponding different scan rates.

The application of electrochemical energy storage is of interest to investigate the electrochemical performance of these np-Ni samples with different morphologies prepared under different magnetic densities (0 mT and 500 mT). Cyclic voltammetry (CV) tests were first employed to characterize their electrochemical capacitance. Figure 2a,b show the typical CV curves at different potential scan rates (5, 10, 20, and 50 mV s^−1^) in 1 M KOH aqueous solution. The potential window was chosen from 0 to 0.55 V. The reversible CV curves are composed of redox peaks, reflecting the Faradic pseudocapacitive nature of the nickel material in alkaline solution. The anodic and cathodic peaks were located at the potentials of 0.50 V and 0.30 V, respectively, associated with the redox reaction of Ni^2+^ to Ni^3+^ at the electrode surface [28,29]. The potential of the anodic peak shifted positively along the potential axial whereas the potential of cathodic peaks shifted negatively when the potential scan rate increased. The increase in the scan rate could enhance the irreversibility of the electrochemical reaction, resulting in the polarization of the electrode and the shift of the location of redox peaks [30].

The average specific capacitance of the np-Ni_0_ and np-Ni_500_ electrodes were then calculated by integrating the CV curves according to the following equation [31]:

(1) $$C=\frac{1}{mv(V_{f}-V_{i})}\int_{V_{i}}^{V_{f}}I(V)dV,$$

where *C* (F g^−1^) is the specific capacitance, *m* (g) is the mass of the active materials of electrode, *v* (V s^−1^) is the scan rate, and *V_i_* and *V_f_* (V) are the initial and final potentials, respectively. *I* (A) is the corresponding current. The CV curves of the np-Ni_500_ electrode showed a larger integrated area than that of the np-Ni_0_ electrode. This indicates that the np-Ni_500_ electrode may exhibit a higher activity of electrochemical energy storage. As shown in Figure 2c, the average specific capacitances of the np-Ni_0_ electrode were calculated as 96, 63, 59, and 40 F g^−1^, while the average specific capacitances of the np-Ni_500_ electrode were calculated as 180, 139, 99, and 57 F g^−1^, at the scan rates of 5, 10, 20, and 50 mV s^−1^, respectively. The specific capacitance decreased gradually with the increasing of the scan rate, which agrees with the literature [32,33]. According to the mathematical relation between specific capacitance (F g^−1^) and specific charge capacity (mAh g^−1^), 1 F g^−1^ = 1 mAh g^−1^ × 3.6 C (mAh g^−1^)^−1^/Δ*V*, where Δ*V* is the potential range for discharge, and the specific charge capacities of the np-Ni_0_ electrode were calculated as 15, 10, 9, and 6 mAh g^−1^ at the scan rates of 5, 10, 20, and 50 mV s^−1^, respectively. The specific charge capacities of the np-Ni_500_ electrode in this study were calculated as 27, 21, 15, and 9 mAh g^−1^, respectively. The larger value of the specific charge capacity indicates more charge accumulated at the np-Ni_500_ electrode surface at the identical measurement conditions. The electrochemical reaction of the np-Ni in KOH electrolyte occurred due to the OH^−^ insertion–desertion [34]. The diffusion of ions from the electrolyte almost made contact with of all the active interface of the electrode at a slower scan rate. When the scan rate was increased, the effective area, as well as the interaction between the ions and the electrode, was reduced due to the nanoporous structure. The insufficient electrochemical reaction leads to a lower specific capacitance and a lower specific charge capacity [35]. Overall, the np-Ni_500_ electrode with better morphology had a larger specific capacitance and larger specific charge capacity than those of the np-Ni_0_ electrode prepared without a magnetic field.

**Figure 3:**
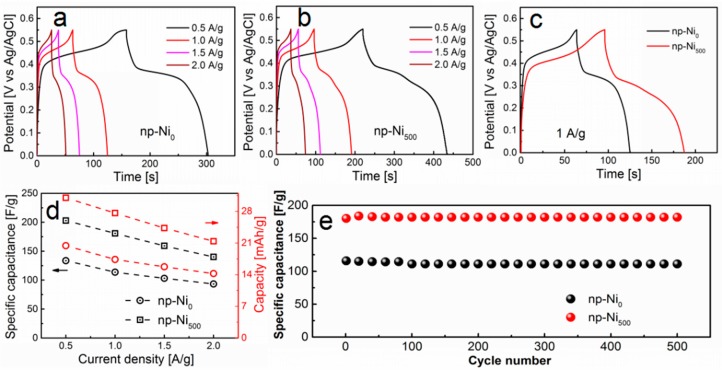
Galvanostatic charge–discharge curves of (**a**) np-Ni_0_ and (**b**) np-Ni_500_. (**c**) Galvanostatic charge–discharge curves of the np-Ni_0_ and np-Ni_500_ electrodes at 1 A g^−1^ current density. (**d**) The specific capacitance at corresponding discharge current densities. (**e**) Cycling performance of the np-Ni_0_ and np-Ni_500_ electrodes at 1 A g^−1^ current density.

Electrochemical energy storage performances of the np-Ni electrodes were further evaluated in galvanostatic charge–discharge measurements. The galvanostatic charge–discharge curves of the np-Ni_0_ and np-Ni_500_ electrodes are shown in Figure 3 at various current densities. The discharge plateaus were located at 0.3–0.4 V, which is the characteristic of pseudo-capacitance or battery behavior, in agreement with the CV measurements. As one example, the comparison in Figure 3c shows that the charge–discharge time of the np-Ni_500_ electrode was larger than that of np-Ni_0_ electrode at 1 A g^−1^ current density, indicating the better energy storage performance of np-Ni_500_. The specific capacitance can be calculated by the following formula [36]:

(2) $$C=\frac{I\Delta t}{m\Delta V},$$

where *C* (F g^−1^) is the specific capacitance, *I* (A) is the discharged current, Δ*t* (s) is the discharged time, and Δ*V* (V) is the potential window for discharge. As plotted in Figure 3d, the specific capacitances of np-Ni_0_ were calculated as 134, 114, 103, and 93 F g^−1^, while the specific capacitances of np-Ni_500_ were calculated as 203, 181, 159, and 140 F g^−1^, at the discharge current densities of 0.5, 1.0, 1.5, and 2.0 A g^−1^, respectively. The specific charge capacities of the np-Ni_0_ electrode were calculated as 20, 17, 16, and 14 mAh g^−1^, while those of the np-Ni_500_ sample were obtained as 30, 28, 24, and 21 mAh g^−1^, at the discharge current densities of 0.5, 1.0, 1.5, and 2.0 A g^−1^, respectively. The specific capacitance and the specific charge capacity of the np-Ni_500_ electrode is much larger than that of the np-Ni_0_ electrode at all current densities in this study. The np-Ni_500_ electrode possesses higher specific capacitance and higher specific charge capacity due to its higher surface area, larger pore size, and better nanoporous structure. This morphology provides more channels for the ions diffusing from the electrolyte to the electrode surface. This increases the effective contact between the ions and the surface, so that the reaction can be completed. Figure 3e shows the cycle stability of the np-Ni_0_ and np-Ni_500_ electrode at the current density of 1 A g^−1^. The specific capacitance loss was about 4% for np-Ni_0_ and 1% for np-Ni_500_ electrodes after 500 cycles.

### 3.2. The Electrochemical Energy Storage Performance of np-Ni under Different Magnetic Fields

**Figure 4:**
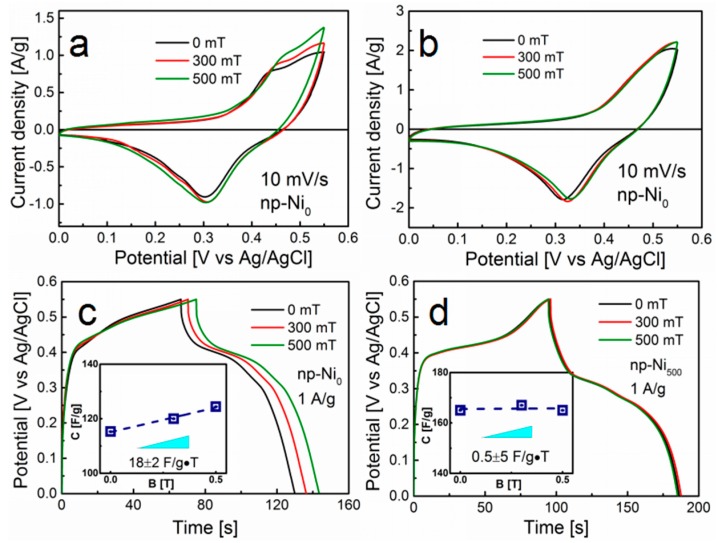
CV curves of the (**a**) np-Ni_0_ and (**b**) np-Ni_500_ electrodes under different magnetic intensities at a 10 mV s^−1^ scan rate. (**c**) Galvanostatic charge–discharge curves of the np-Ni_0_ and (**d**) np-Ni_500_ electrodes under different magnetic intensities at 1 A g^−1^ current density. Insets in (**c**) and (**d**) are the specific capacitance curves as a function of magnetic intensity corresponding to the np-Ni_0_ and np-Ni_500_ electrodes.

It can be seen that the external magnetic field can influence the morphology of np-Ni, leading to the enhancement of electrochemical energy storage performance. Since Ni metal is a ferromagnetic material, we find that it is of interest to further investigate the effect of the external magnetic field on the electrochemical process of these two different np-Ni electrodes. The effect of the external magnetic field with different flux densities on CV measurement is presented in Figure 4a,b at the scan rate of 10 mV s^−1^ for both np-Ni_0_ and np-Ni_500_. The peak currents of the np-Ni_0_ electrode in Figure 4a slightly increased with increasing the magnetic flux densities. The CV curves of the np-Ni_500_ electrode in Figure 4b were weakly dependent on the magnetic flux densities. In order to support the observation from the CV data, the effect of the external magnetic field on the electrochemical energy storage performance was further studied by charge–discharge curves. For np-Ni_0,_ shown in Figure 4c, the charge–discharge time increased with the applied magnetic field from 0 mT to 500 mT at the same current density of 1 A g^−1^. The specific capacitances of np-Ni_0_ were calculated as 115 F g^−1^, 120 F g^−1^, and 125 F g^−1^ under 0 mT, 300 mT, and 500 mT, respectively. The change in the specific capacitance was plotted as a function of the magnetic flux density and is presented in the inset of Figure 4c. The positive slope value (18 ± 2 F g^−1^ T^−1^) indicates that the specific capacitance can be enhanced by applying a magnetic field. The additional galvanostatic charge–discharge curves for the np-Ni_500_ electrode were indeed independent of the magnetic flux densities (Figure 4d). The specific capacitance maintained a value of 165 F g^−1^. The specific charge capacities are available in Supplementary Materials (see Figure S2).

**Figure 5:**
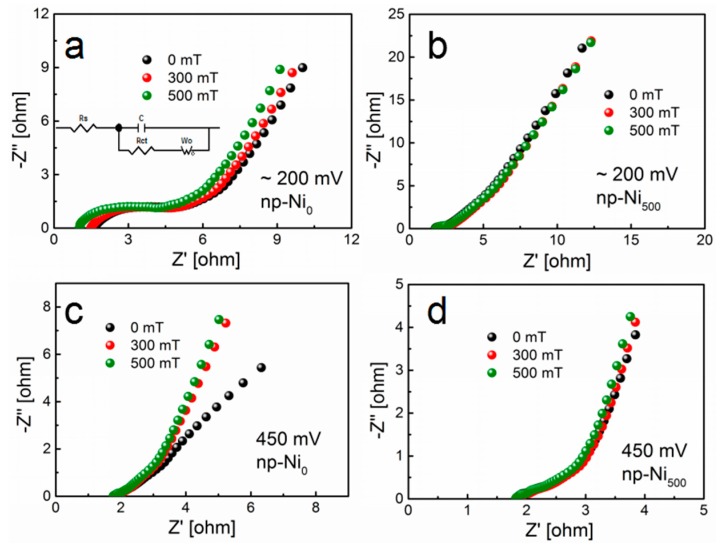
Nyquist plots of the np-Ni_0_ and np-Ni_500_ electrodes under different magnetic flux densities at an open-circuit potential ((**a**) np-Ni_0_, (**b**) np-Ni_500_) and 450 mV ((**c**) np-Ni_0_, (**d**) np-Ni_500_) over the frequency range of 10 kHz to 0.1 Hz.

In order to understand the difference between np-Ni_0_ and np-Ni_500_, we investigated the EIS behaviors under different magnetic fields. The performance of EIS for the electrodes at an open-circuit potential (~200 mV) or over the frequency range of 10 kHz to 0.1 Hz with the potential amplitude of 5 mV are shown in Figure 5a,b. The impedance plots are composed of the electrolyte solution resistance (*R*_s_) and the charge transfer resistance (*R*_ct_) at a high frequency region and the leakage resistance (*R*_leak_) at a low frequency region [37,38]. Figure 5a shows the performance of EIS for the np-Ni_0_ electrode at an open-circuit potential under the magnetic flux densities of 0, 300, and 500 mT. The corresponding simulated results of the electric impedance spectra are summarized in Table 1. With increase of the magnetic flux densities, the values of *R*_s_ and *R*_ct_ decreased. This indicates that the convection in the bulk electrolyte was enhanced and the electrode possessed a lower charge transfer resistance under the effect of the magnetic field, leading to the increase of the specific capacitance [39]. The increase of *R*_leak_ may be due to the enhancement of electrolyte convection, which made more ions come into contact with the interface to form more double layers [21,37]. For the case of the np-Ni_500_ electrode in Figure 5b, the resistance of the electrodes was weakly dependent on the magnetic flux densities. To further study the effect of the magnetic field on the pseudo-capacity process, we tested the impedance at 0.45 V under different magnetic flux densities, as shown in Figure 5c,d. The rate of the diffusion increased as the slope of the line increased. This result further indicates that the magnetic field can improve the mass transport process of the ions for the np-Ni_0_ electrode, whereas the magnetic field has little influence on the np-Ni_500_ electrode. The EIS data support our experimental data above.

This phenomenon could be attributed to the Lorentz force, which acts on the movement of ions in a perpendicular magnetic field (magnetohydrodynamic phenomenon). The morphology of the np-Ni_0_ electrode presented a particle-stacking characteristic. We compared the morphologies of the np-Ni_0_ electrode before and after the electrochemical measurement under the magnetic field (see Figure S3, Supplementary Materials). The whole structure was not changed under the effect of the magnetic field. For such a structure of np-Ni_0_, the ions only came into contact with the geometrical surface of the electrode and it was difficult to reach the inside region of the nanoporous electrode. When applying an external magnetic field, the ions in electrolyte changed their original routine to reach the greater specific surface area of the nanoporous electrode (see Figure S4, Supplementary Materials). The electrolyte convection was promoted in the bulk electrolyte and led to the reduction of *R*_s_ [6,40]. The exposed electrode surface area was enhanced with the increase of the magnetic field, which caused the reduction of *R*_ct_ [41,42]. The increase of the electrode surface built up a double layer and accelerated the rate of diffusion. Generally, the electrochemical performance of the np-Ni_0_ electrode was improved with the application of an external magnetic field. For the np-Ni_500_ electrode, the influence was not obvious due to its high surface area and large pore size.

## 4. Conclusions

In summary, the electrochemical performance of np-Ni_500_ as an electrode material for supercapacitors, which was prepared under a magnetic field, is much better than that of normal np-Ni_0_, which was prepared without a magnetic field. The electrochemical capacitance of the np-Ni_0_ electrode was further enhanced with the magnetic flux density. For the np-Ni_0_ electrode, the electrolyte solution (*R*_s_) and the charge transfer resistance (*R*_ct_) decreased when applying the magnetic field. The magnetic field can accelerate the diffusion rate to improve the capacitance. These findings present a potential revolution of traditional electrochemical capacitors by simply applying an external magnetic field.

## Figures and Tables

**Table 1:** Simulated results of electric impedance spectra of np-Ni_0_ at an open-circuit potential under different magnetic flux densities.

B (mT)	Simulated Internal Resistance (Ohm)		
-	*R*_s_	*R*_ct_	*R*_leak_
0	1.62	1.96	20.70
300	1.44	1.80	21.52
500	1.03	1.66	23.10

## Supplementary Materials

The following are available online at https://www.mdpi.com/2079-4991/9/5/694/s1, Figure S1: The pore size distributions of np-Ni_0_ and np-Ni_500_, Figure S2: Specific capacitance curves as a function of magnetic intensity corresponding to np-Ni_0_ (a) and np-Ni_500_ (b) electrodes, Figure S3: The SEM image of np-Ni_0_ after the electrochemical measurement under a 500 mT magnetic field, Figure S4: Schematic illustration of ion transportation before and after applying a magnetic field.
