# Supplementary material for: The Effect of an External Magnetic Field on the Electrochemical Capacitance of Nanoporous Nickel for Energy Storage
Source: Nanomaterials (Basel). 2019 May 4;9(5):694. doi: 10.3390/nano9050694 (PMC6566679; doi:10.3390/nano9050694)
Supplement: Supplementary file 1 [file nanomaterials-09-00694-s001.pdf]

Supporting Information

The Effect of an External Magnetic Field on the Electrochemical Capacitance of Nanoporous Nickel for Energy Storage

Haixia Zhang [1], Zhifei Han [1] and Qibo Deng [1,*]

[1] Tianjin Key Laboratory of Advanced Functional Porous Materials, Institute for New Energy Materials and Low-Carbon Technologies, School of Materials Science and Engineering, Tianjin University of Technology, Tianjin 300384, China.; haixia0202@126.com (H.Z.); 183124331@stud.tjut.edu.cn (Z.H.)

* Correspondence: qibodeng@tjut.edu.cn; Tel.: +86-022-6021-5388 (Q.D.)

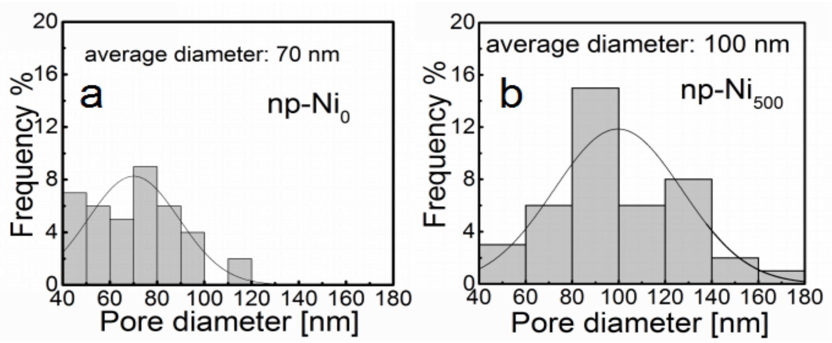

Figure S1. The pore size distributions of np-Ni_0 and np-Ni_{500}.

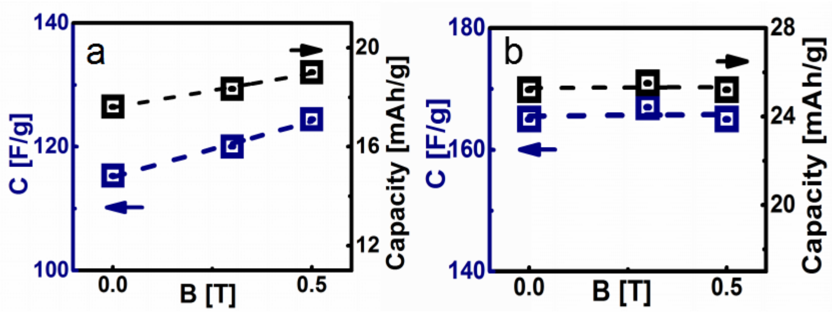

Figure S2. Specific capacitance curves as a function of magnetic intensity corresponding np-Ni_0 (a) and np-Ni_{500} (b) electrodes.

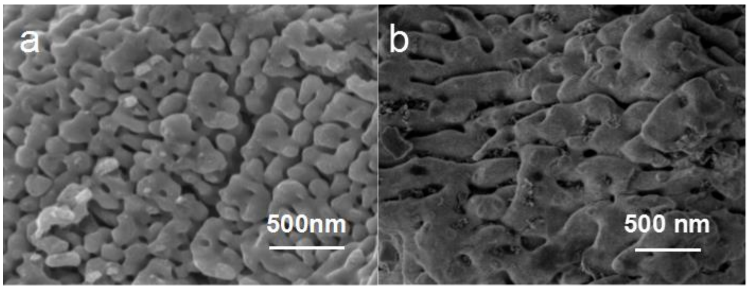

Figure S3. The SEM image of the np-Ni_0 after the electrochemical measurement under the 500 mT magnetic field.

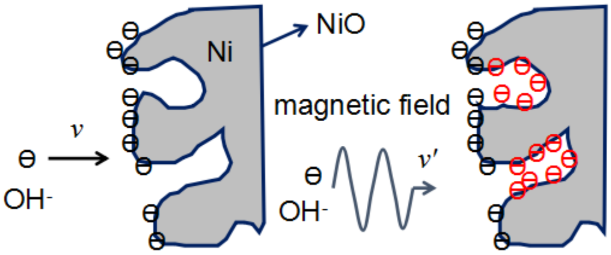

Figure S4. Schematic illustration of ion transportation before and after applying a magnetic field. (adapted from ref. Nano Energy, 2014, 6, 180-192).
